# Unravelling the determinants of life expectancy during and after the COVID-19 pandemic: a qualitative comparative analysis

**DOI:** 10.7189/jogh.15.04126

**Published:** 2025-03-28

**Authors:** Xiao Li, Jialing Zhu, Jae Man Park, Jordan Mitchell

**Affiliations:** 1 Department of Management, University of Nebraska at Kearney, Kearney, Nebraska, USA; 2 Georgetown University, Ralph Lauren Center for Cancer Prevention, Lombardi Comprehensive Cancer Center, Washington DC, USA; 3 Department of Management, University of Texas, Health Science Center at Houston, Policy & Community Health, Houston, Texas, USA; 4 Department of Healthcare Administration, University of Houston Clear Lake, Houston, Texas, USA

## Abstract

**Background:**

Disparities in life expectancy persist across countries, despite overall improvements in recent years. The COVID-19 pandemic further exacerbated these disparities. While substantial research has investigated life expectancy determinants, the factors driving variations across countries remain insufficiently explored.

**Methods:**

This study innovatively employed Qualitative Comparative Analysis with data from 2020–2022, integrating multiple global data sources. We examined the complex causal patterns among conditions, including educational attainment, economic prosperity, environmental quality, social stability, urban development, and public health capacity within a case-oriented framework. Variables were calibrated into fuzzy sets to analyse necessary and sufficient conditions, with intermediate results tested across cases per solution, including robustness tests to validate the findings.

**Results:**

Environmental quality, represented by access to electricity, consistently emerged as a necessary and sufficient condition across seven key case scenarios for achieving high life expectancy. Each case highlights unique pathways that align with different combinations of socioeconomic and policy conditions, illustrating that diverse approaches can lead to positive outcomes. In addition to environmental quality, factors such as mean years of schooling, gross national income per capita, urban population density, and measles immunisation were found to be influential in various combinations within these cases, underscoring the complexity of life expectancy determinants.

**Conclusions:**

Our findings indicate that while core determinants like environmental quality are crucial, countries can enhance life expectancy through unique, context-dependent pathways that integrate environmental, educational, economic, and public health factors. Specifically, countries may focus on different policy areas based on their socio-economic conditions and development priorities to optimise life expectancy outcomes.

Life expectancy, refers to life expectancy at birth in this study, is a fundamental indicator of population health [1,2]. Between 1995–2017, the global average life expectancy rose from 66.20 to 72.89 years, demonstrating an overall upward trend. However, disparities in life expectancy persist across countries [3,4]. According to Liou et al., approximately 34% of the variability in life expectancy improvement can be explained by country-level differences, based on an analysis of 201 countries between 1950–2015 [5].

Recent data has shown further fluctuations in global life expectancy, with a notable decline of 0.92 years from 2019–2020 and a further 0.72-year reduction from 2020–2021 [6]. While disparities in life expectancy between countries became more pronounced during this period [7–9], these divergences reflect long-standing inequities. Despite extensive research, the underlying factors driving variations in life expectancy across countries remain insufficiently explored. Identifying these key determinants is essential for understanding global health trends and developing effective strategies to address disparities in life expectancy over the long term.

Research consistently demonstrates that socioeconomic factors, such as education attainment, economics prosperity, environmental quality, social stability, urban development, and public health capacity, play pivotal roles in determining life expectancy. Education attainment, in particular, has been shown to have a positive association with life expectancy [10–13]. Meara and Richards found that gains in life expectancy at age 25 have predominantly occurred among those with higher educational attainment, except for Black males. This trend has led to a 30% increase in the disparity in life expectancy between more and less educated groups [11]. A recent systematic review and meta-analysis analysed, covering data from 70 locations across 59 countries and yielding 10 355 observations [12]. The study found a clear dose: response relationship between education and all-cause adult mortality, showing that each additional year of education was associated with an average 1.9% reduction in mortality risk [12]. Economics prosperity is widely recognised as key determinants of life expectancy. For example, higher economic status, often measured by gross national income (GNI) or gross domestic product *per capita*, was strongly associated with better life expectancy outcomes [14–16].

Environmental quality, such as access to essential public resources and infrastructure, like clean water, electricity, or internet, have also been shown to contribute significantly to life expectancy [13,17,18]. Additionally, annual population growth serves as a crucial environmental factor influencing life expectancy. For instance, Popoola’s Granger causality analysis indicated that population growth could significantly lower life expectancy in Nigeria, with findings significant at the 10% level [19].

Social stability, represented by employment ratio, for example, was also identified to be related to life expectancy. For instance, an analysis of 2015–2017 German data revealed that a 2.47% difference in unemployment rates between two western districts was associated with a 0.6-year reduction in life expectancy in the district with higher unemployment [20]. Urban development, measured by the urban population ratio, showed a mixed impact on life expectancy. On one hand, less overcrowded urban areas tended to have longer life expectancies [13], while on the other hand, rural populations experienced a decline in life expectancy [21,22].

Public health capacity factors, such as annual population growth, immunisation rates, human immunodeficiency virus (HIV) prevalence, and other health index, are strongly linked to life expectancy. Van Wijhe et al. conducted a historical time series analysis, revealing rising measles vaccination coverage was associated with the declining overall mortality rates [23]. Neumayer highlighted that the HIV/AIDS epidemic disrupted the global trend toward reducing disparities in life expectancy. The inequality reduction achieved by 1985 was not expected to return before 2015, and if the epidemic worsened, this divergence could persist until 2050, further delaying progress in global life expectancy equality [24].

While existing studies provide valuable insights, more research is needed to explore how key determinants of life expectancy interact, particularly considering the recent global event, the COVID-19 pandemic. Grounded in the Determinants of Health Framework, which emphasises that health outcomes, including life expectancy, are shaped by a complex interplay of social, economic, environmental, and health care-related factors [25], this study aims to examine the determinants and their interaction effects on life expectancy from 2020–2022. By focusing on short-term drivers during and immediately following the pandemic, our study also draws implications for longer-term trends. Using the Qualitative Comparative Analysis (QCA) method, we aim to identify enduring influences across nations. To our best knowledge, this is the first study to explore life expectancy during the pandemic using QCA to uncover configurational effects.

## METHODS

### Data sources and sample size

This study sourced from three global data sets: the Human Development Indicators from the United Nations Development Programme, the World Development Indicators from the World Bank Open Data, and the COVID-19 Containment and Health Index from the Our World in Data, to explore the determinants of life expectancy across countries during the years 2020–2022. This specific three-year time frame was chosen to capture the unique global impact of the COVID-19 pandemic on life expectancy. The data sets were merged using country names to align data points. Initially, the sample size from the World Development Indicators data set was 271 countries per year. After removing five unassigned countries, the sample size was reduced to 266. For the COVID-19 Containment and Health Index data, the initial sample consisted of 202 760 observations. We calculated the average index value for each country by year, resulting in a sample size of 185 countries per year. After merging the World Development Indicators with the averaged COVID-19 Containment and Health Index data, we obtained a merged data set with 183 observations per year. The Human Development Composite Indices data set initially contained 206 countries per year. After merging with the combined data set from the World Development Indicators and the COVID-19 Containment and Health Index, the sample size was reduced to 175 countries per year. Ultimately, after removing the missing data, we obtained a complete data set of 130 countries per year. For detailed information on the data derivation process, please see the **Online Supplementary Document**. Institutional Review Board approval was waived due to the use of secondary data.

### Measures

#### Dependent variable

In this study, our dependent variable is life expectancy (at birth), which refers to ‘the average number of years that a newborn could expect to live, if he or she were subject to the age-specific mortality rates of a given period’ [26]. Life expectancy reflects mortality rates and serves as an indicator of overall health conditions [1]. The data points are derived from the Human Development Index provided by the United Nations Department of Economic and Social Affairs.

#### Independent variable

Our independent variables, or conditions, include indicators related to education attainment, economics prosperity, environment quality, social stability, urban development, and public health capacity.

1. Education attainment: education is represented by the mean years of schooling, which indicates ‘the average number of completed years of education for a country’s population, excluding any years spent repeating grades’ [27]. The data points for education are derived from the Human Development Index, provided by the United Nations Educational, Scientific, and Cultural Organization. These estimates cover the population aged 25 years and older [28].

2. Economic prosperity: gross national income *per capita* represents a country’s economic output, expressed in USD using the World Bank Atlas method and divided by the midyear population [27]. This measure includes the value added by all resident producers, product taxes (minus subsidies), and net income from abroad. To reduce price and exchange rate fluctuations, the Atlas method averages exchange rates from the current and two preceding years, adjusted for inflation differences between the country and major economies like the Euro area, Japan, the UK, and USA [27]. The data point sources from LAC Equity Laboratory Tabulations of the World Development Indicators of the World Bank Open Data platform.

3. Environmental quality: environment factors include two indicators:

1) access to electricity (% of population), an indicator of a country’s environmental infrastructure, refers to the percentage of the population with electricity access [27]. The electrification data are sourced from industry reports, national surveys, and international databases [27], and is compiled by the World Bank’s Global Electrification Database and the World Development Indicators.

2) annual population growth rate. Population, defined using the *de facto* approach, which includes all residents regardless of legal status or citizenship, is measured by its annual growth, calculated as the exponential growth rate of the midyear population from the previous year to the current year, expressed as a percentage [27]. Population growth serves as an indicator of health because it reflects the broader demographic and social conditions that affect public health. This data are initially gathered and refined by the United Nations Population Division.

4. Social stability: the employment-to-population ratio, representing social stability, refers to the proportion of a country’s working-age population (15 years and older) that is employed [27]. Employment includes individuals engaged in any activity to produce goods or services for pay or profit, even if temporarily absent from work [27]. This ratio reflects how effectively an economy provides jobs [27]. The data are sourced from the International Labor Organization of the World Bank’s World Development Indicators.

5. Urban development: urban population (% of total population), an indicator of urban development, represents the percentage of individuals living in urban areas, defined by national statistical offices, per 100 people in the total population [27]. The data are collected and refined by the United Nations Population Division and featured in the World Bank’s World Development Indicators.

6. Public Health Capacity: we include three health-related indicators:

1) measles immunisation rate – a key indicator of advancing human health, represents the percentage of children aged 12–23 months who received the measles vaccine either before their first birthday or at any time prior to the survey [27]. A child is considered fully immunised after receiving a single dose of the vaccine [27]. This data are collected and refined by the World Health Organization and The United Nations Children's Fund.

2) HIV prevalence – refers to the percentage of individuals aged 15–49 who are infected with HIV in each country’s population. It represents the vulnerability of this population and the potential risk of the virus spreading to the broader community [27]. This data are collected and refined by the Joint United Nations Programme on HIV/AIDS.

3) COVID-19 Containment and Health Index – ranging from 0 to 100 (with 100 being the strictest), is a composite measure based on 13 policy indicators such as school closures, travel bans, testing, and vaccine policies [29]. Sourced from the University of Oxford, this index is crucial for understanding how varying levels of pandemic responses across countries may have influenced life expectancy.

The first two indicators are sourced from the World Bank’s World Development Indicators, while the third comes from our Our World in Data.

### Statistical analyses

We employed QCA to identify complex causal patterns among multiple conditions in a case-oriented framework [30]. This method is particularly valuable for identifying configurations of conditions that are necessary or sufficient for life expectancy outcomes, going beyond the additive and linear assumptions of regression models. In addition, the use of QCA was further motivated by the sample size of this study. With 130 observations per year, traditional regression methods might lack the statistical power to identify complex interactions between variables. By contrast, QCA is designed to analyse small- to medium-sized data sets, enabling the identification of configurational effects across diverse cases.

Variables were calibrated into either fuzzy sets, which allow for varying degrees of membership between full non-membership and full membership, or crisp sets [31]. This calibration approach enhances the robustness and validity of the QCA findings by accurately capturing the nuanced influence of each condition on the outcome of life expectancy (appendix 1in the **Online Supplementary Document**). The analysis further includes both necessary and sufficient condition assessments (configuration analyses), to elucidate the complex interplay among our measures. The necessity analysis assesses whether any of the conditions were required for the outcome to occur [32], and the degree of necessity was measured using two criteria: consistency and coverage. Consistency measures how often the independent variable is present when the outcome (life expectancy) occurs coverage indicates the extent to which the condition explains the outcome, with higher coverage meaning a larger portion of cases is explained [33]. A consistency threshold of 0.9 and a coverage threshold of 0.5 suggest that the condition may be necessary for the outcome [33]. Based on the results of the necessity analysis, we decided if we perform a sufficiency analysis to identify sets of configurations that were always present when the desired outcome occurred. Additionally, we conducted robustness checks to confirm the main findings, utilising the methods developed by Oana et al. [34].

All analyses were performed with the *R* Programming software (R Foundation, Boston, MA, USA, Version 4.3.2) with packages ‘QCA 3.22’ and ‘SetMethods 4.0.’

## RESULTS

### Descriptive statistics

The sample characteristics for this study comprisies 130 observations for each year (2020, 2021, and 2022), for a total of 390 observations (**Table 1**). Over the three-year period, variables such as GNI *per capita*, access to electricity, and the COVID-19 Containment and Health Index exhibit high variability, reflecting notable differences across countries. In contrast, life expectancy at birth, mean years of schooling, annual population growth, employment-to-population ratio, urban development, immunisation rates, and HIV prevalence show relatively low variability, indicating stability in these areas during the examined period.

**Table 1:** Country descriptive statistics in 2020–2022

Variable	Year				
		**2020 (n = 130)**	**2021 (n = 130)**	**2022 (n = 130)**	**Total (n = 390)**
**Dependent variable**					
Life expectancy	Min/max	52.8 / 83.4	52.5 / 83.8	53.0 / 84.1	52.5 / 84.1
	MD (IQR)	71.7 (64.8–75.4)	70.4 (63.8–74.2)	71.3 (64.8–75.8)	71.1 (64.5–75.3)
	Mean (SD)	70.4 (7.2)	69.7 (7.3)	70.5 (7.6)	70.2 (7.3)
**Independent variables/conditions**					
Mean years of schooling	Min/max	1.3 / 13.8	1.3 / 13.8	1.3 / 13.8	1.3 / 13.8
	MD (IQR)	8.6 (5.7–10.7)	8.6 (5.7–10.7)	8.6 (5.7–10.7)	8.6 (5.7–10.7)
	Mean (SD)	8.2 (3.2)	8.3 (3.2)	8.3 (3.2)	8.3 (3.2)
Gross national income *per capita*	Min/max	716.0 / 87 000	707.3 / 92 000	690.7 / 96 000	690.7 / 96 000
	MD (IQR)	9446.4 (3700–19 000)	9528.0 (3600–21 000)	9995.6 (3600–22 000)	9702.2 (3600–20 000)
	Mean (SD)	15 000 (16 000)	16 000 (17 000)	16 000 (18 000)	16 000 (17 000)
Access to electricity (% of population)	Min/max	7.3 / 100.0	7.7 / 100.0	8.4 / 100.0	7.3 / 100.0
	MD (IQR)	98.7 (64.3–100)	99.3 (66.1–100]	99.8 (68.4–100)	99.4 (65.4–100)
	Mean (SD)	81.2 (27.8)	81.8 (27.3)	82.2 (26.8)	81.7 (27.2)
Population growth (annual %)	Min/max	−0.57	−1.16	−1.68	−1.68
	MD (IQR)	1.4 (0.4–2.2)	1.2 (0.4–2.1)	1.2 (0.5–2.2)	1.2 (0.5–2.2)
	Mean (SD)	1.3 (1.2)	1.1 (1.3)	1.2 (1.3)	1.2 (1.3)
Employment to population ratio	Min/max	26.4 / 87.5	26.0 / 87.5	26.9 / 88.5	26.0 / 88.5
	MD (IQR)	55.1 (48.7–62.5)	56.1 (49.1–63.3)	57.6 (49.9–64.6)	56.5 (49.3–63.4)
	Mean (SD)	55.2 (11.6)	55.8 (11.8)	56.9 (11.7)	56.0 (11.7)
Urban population (% of total population)	Min/max	13.3 / 100.0	13.5 / 100.0	13.6 / 100.0	13.3 / 100.0
	MD (IQR)	57.2 (38.0–75.8)	57.8 (38.6–76.3)	58.2 (39.3–76.7)	57.6 (38.5–76.4)
	Mean (SD)	57.3 (22.7)	57.7 (22.7)	58.0 (22.6)	57.7 (22.6)
Immunisation, measles (% of children ages 12–23 mo)	Min/max	41.0 / 99.0	36.0 / 99.0	37.0 / 99.0	36.0 / 99.0
	MD (IQR)	88.0 (76.2–94.0)	88.0 (74.2–94.0)	88.0 (74.2–95.0)	88.0 (75.2–94.8)
	Mean (SD)	83.5 (13.9)	82.5 (15.0)	83.1 (14.6)	83.0 (14.5)
Prevalence of HIV	Min/max	0.1 / 27.6	0.1 / 26.8	0.1 / 25.9	0.1 / 27.6
	MD (IQR)	0.4 (0.1–1.1)	0.4 (0.1–1.1)	0.4 (0.1–1.0)	0.4 (0.1–1.1)
	Mean (SD)	1.8 (4.2)	1.7 (4.1)	1.7 (3.9)	1.7 (4.0)
COVID-19 containment and health index	Min/max	12.6 / 62.7	17.3 / 77.3	16.4 / 63.6	12.6 / 77.3
	MD (IQR)	48.3 (39.4–53.6)	58.3 (50.5–65.7)	36.5 (31.2–44.2)	47.2 (36.9–56.3)
	Mean (SD)	46.5 (9.6)	56.6 (12.8)	37.4 (9.2)	46.9 (13.2)

IQR – interquartile range, MD – median, Min/max – minimum/maximum, n – number, SD – standard deviation

### QCA results

The calibration thresholds for both the dependent and independent variables/conditions used in QCA are presented in Table S1 in the **Online Supplementary Document**. The calibration was based on three key thresholds: full membership (0.95, representing the 95th percentile), crossover point (0.5, representing the 50th percentile), and full non-membership (0.05, representing the 5th percentile). These thresholds were applied to transform raw data into fuzzy set scores for analysis.

**Figure 1:**
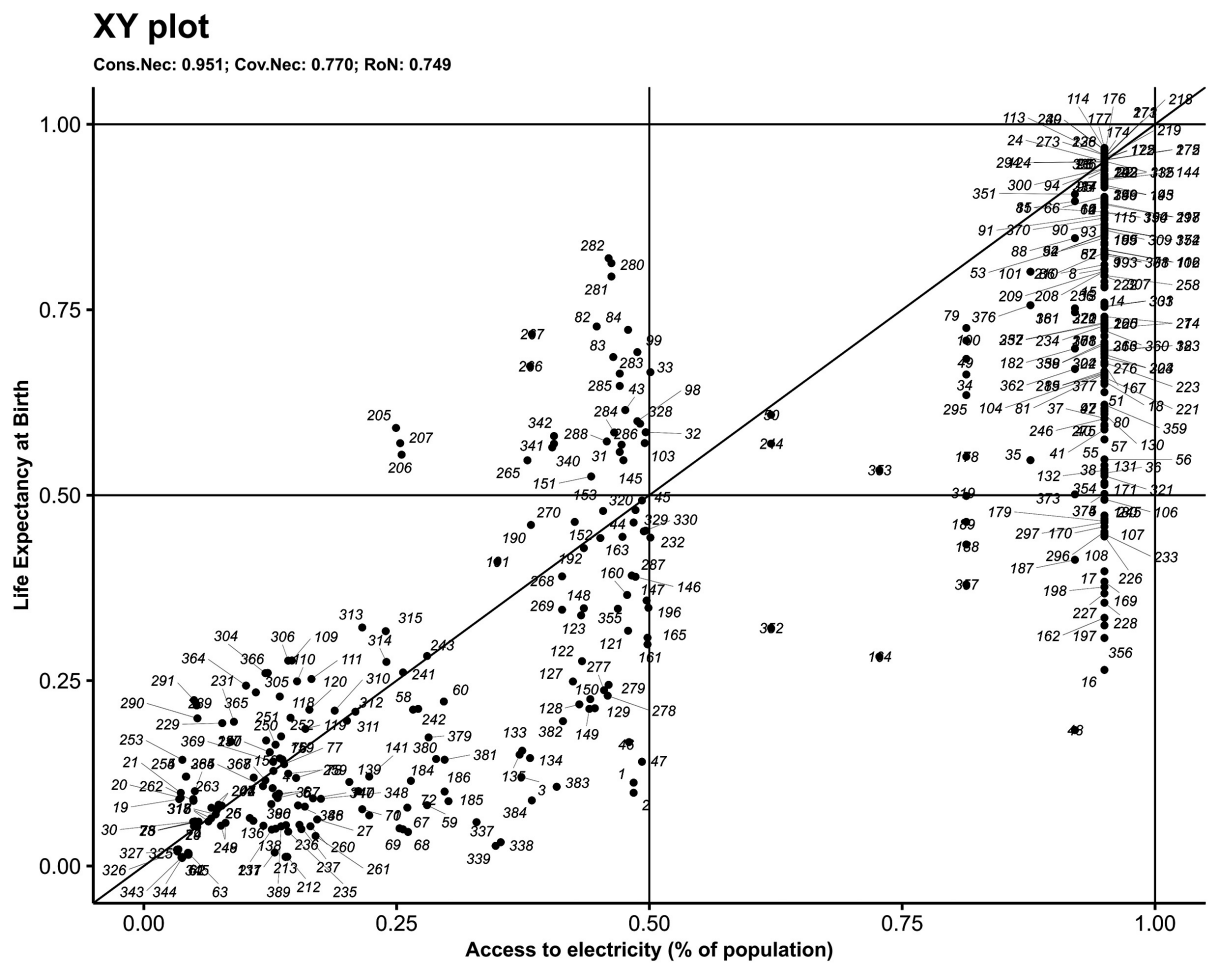
QCA necessary analysis plot: access to electricity as necessary. QCA – qualitative comparative analysis.

As shown in **Table 2**, access to electricity emerged as a highly consistent necessary condition for high life expectancy, with a consistency score of 0.95, surpassing the commonly accepted threshold of 0.9. This suggests that access to electricity is a crucial factor in leading to high life expectancy across the cases examined in our study. We further analysed access to electricity as a necessary condition for life expectancy, as shown in **Figure 1**, where the relevance of necessity is 0.749 exceed the threshold of 0.5. Relevance of necessity is another indicator of necessity, which balances consistency and coverage, evaluating whether a condition is both necessary and meaningful, while adjusting for cases where high consistency might be trivial in the data set [35]. These metrics collectively support that access to electricity is a key contributor for achieving high life expectancy across the observed regions: a strong positive correlation between access to electricity and life expectancy. Several other conditions approached this threshold, indicating their importances in the outcome. Specifically, mean years of schooling showed a consistency level of 0.84, while GNI *per capita* and urban population percentage both had consistencies of 0.82, and the measles immunisation rate scored 0.81. Though these conditions did not meet the strict 0.9 threshold, their proximity underscores their relevance in contributing to high life expectancy. Furthermore, all these conditions demonstrated coverage levels exceeding 0.5, indicating that each one covers a significant portion of the outcome cases. Specifically, GNI *per capita* had the highest coverage at 0.90, followed by mean years of schooling at 0.80, urban population percentage at 0.78, and immunisation rate at 0.74.

**Table 2:** QCA analysis of necessary conditions results

Independent variables	Life expectancy				~ *Life expectancy			
	**Consistency**	**Coverage**	**BECONS distance**	**WICONS distance**	**Consistency**	**Coverage**	**BECONS distance**	**WICONS distance**
Mean years of schooling	0.84	0.80	0.02	0.06	0.48	0.50	0.07	0.72
~ *Mean years of schooling	0.48	0.46	0.02	0.13	0.81	0.84	0.09	0.33
Gross national income *per capita*	0.82	0.90	0.03	0.07	0.41	0.49	0.10	0.55
~ *Gross national income *per capita*	0.53	0.46	0.03	0.11	0.91	0.84	0.08	0.25
Access to electricity (% of population)	0.95	0.77	0.01	0.04	0.50	0.44	0.09	0.52
~ *Access to electricity (% of population)	0.31	0.36	0.12	0.18	0.74	0.94	0.28	0.16
Population growth (annual %)	0.53	0.49	0.04	0.11	0.83	0.82	0.13	0.22
~ *Population growth (annual %)	0.81	0.81	0.04	0.06	0.49	0.53	0.12	0.43
Employment to population ratio	0.63	0.61	0.06	0.10	0.67	0.70	0.08	0.48
~ *Employment to population ratio	0.69	0.66	0.07	0.09	0.63	0.65	0.10	0.38
Urban population (% of total population)	0.82	0.78	0.02	0.07	0.50	0.51	0.05	1.06
~ *Urban population (% of total population)	0.48	0.47	0.02	0.13	0.78	0.82	0.09	0.35
Immunisation, measles (% of children 12–23 mo)	0.81	0.74	0.01	0.07	0.55	0.54	0.10	0.41
~ *Immunisation, measles (% of children 12–23 mo)	0.50	0.51	0.03	0.12	0.74	0.81	0.06	0.57
Prevalence of HIV	0.42	0.53	0.02	0.15	0.63	0.85	0.05	1.00
~ *Prevalence of HIV	0.89	0.69	0.02	0.05	0.66	0.55	0.02	1.47
COVID-19 containment and health index	0.68	0.66	0.48	0.07	0.59	0.62	1.51	0.02
~ *COVID-19 containment and health index	0.61	0.58	0.59	0.07	0.68	0.70	1.65	0.02

BECONS distance – best consistency distance, WICONS distance – worst-in consistency distance

*Absence of a condition.

The intermediate results and corresponding cases for each solution are shown in **Table 3**. The analysis identified seven distinct pathways leading to the outcome (life expectancy at birth), with each pathway representing a unique combination of explanatory conditions. These pathways reflect different causal mechanisms through which the outcome (high life expectancy at birth) is achieved. The core and peripheral conditions are visually distinguished by the size of the icons in the table. Core conditions, marked with larger solid icons ‘●’, are those that appear in both the intermediate and parsimonious solutions, indicating that they are more directly and consistently associated with the outcome (high life expectancy at birth) across various pathways. In contrast, peripheral conditions, represented with smaller solid icons ‘●’, are present only in the intermediate solution and may play a supporting role but are less critical to explaining the outcome (high life expectancy at birth). The icons of ‘⊗’ indicate the condition is absent in the specific configuration but still contributes to achieving the outcome (high life expectancy at birth). Each of the seven pathways showed consistency scores above 0.8, ranging from 0.957 to 0.983, indicating they are individually sufficient and reliable in predicting high life expectancy at birth. While access to electricity is a central driver, education and economic factors remain critical, and the importance of other factors, such as measles immunisation rates, varies depending on the specific context.

**Table 3:** QCA analysis of necessary conditions results

Independent variables	Life expectancy						
	**Configuration1**	**Configuration2**	**Configuration3**	**Configuration4**	**Configuration5**	**Configuration6**	**Configuration7**
Mean years of schooling	●*	●*	●*	●*	●*	⊗†	●*
Gross national income *per capita*	●*	●*	●*	●*	●*	●*	●*
Access to electricity (% of population)	●‡	●‡	●‡	●‡	●‡	●‡	●‡
Population growth (annual %)	⊗†		⊗†	⊗†	⊗†	⊗†	⊗†
Employment to population ratio	⊗†	●*	●*	⊗†	⊗†	●*	●*
Urban population (% of total population)	●*	●*	●*		⊗†	⊗†	●*
Immunisation, measles (% of children ages 12–23 mo)	●*	●*	⊗†	●*	⊗†	●*	●*
Prevalence of HIV		⊗†	●*	⊗†	⊗†	⊗†	
COVID-19 containment and health index				●*	⊗†	●*	⊗†
PRI	0.916	0.953	0.747	0.939	0.868	0.8	0.913
Raw coverage	0.443	0.448	0.24	0.383	0.205	0.207	0.383
Unique coverage	0.007	0.061	0.021	0.012	0.006	0.016	0.009
BECONS distance	0.011	0.018	0.011	0.015	0.004	0.015	0.011
WICONS distance	0.095	0.087	0.058	0.095	0.058	0.066	0.058
Solution consistency				0.956			
Solution PRI				0.909			
Solution coverage				0.632			

BECONS – best consistency, PRI – proportional reduction in inconsistency, QCA – qualitative comparative analysis, WICONS – worst-in consistency

*Peripheral condition (present).

†Condition absent.

‡Core condition (present).

**Figure 2:**
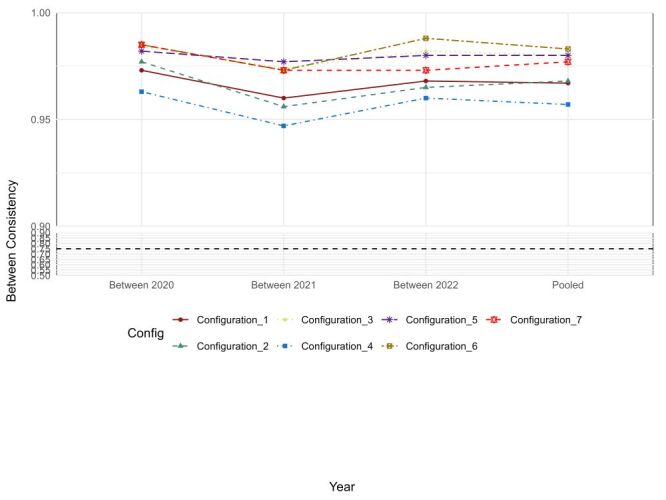
QCA BECONS plot: life expectancy. BECONS – best consistency, QCA – qualitative comparative analysis.

Raw coverage measures the proportion of cases in the outcome set that can be explained by each pathway, with values ranging from 0.205 (Configuration 5) to 0.448 (Configuration 2). This indicates that Configuration 2, for example, accounts for 44.8% of the cases where high life expectancy is observed. Unique coverage reflects the proportion of cases that can only be explained by a particular pathway, excluding overlaps with other configurations. For instance, Configuration 2 has a unique coverage of 0.061, meaning that 6.1% of the cases are uniquely explained by this specific pathway, without being covered by any other configuration (**Table 3**; **Figure 2**).

Additionally, we assessed the reliability and validity of the seven pathways using three key measures: solution consistency, solution coverage, and solution Proportional Reduction in Inconsistency (PRI). Solution consistency reflects how consistently the full set of solutions leads to the desired outcome. A threshold of 0.8 is generally recommended, regardless of sample size. As shown in **Table 3**, the solution consistency of 0.956 indicates a strong and consistent relationship between the pathways and the outcome. Solution coverage, on the other hand, measures the extent to which the identified pathways account for the observed cases. With a solution coverage of 0.632, our results suggest that the seven pathways jointly explain 63.2% of all cases. While there is no standard threshold for solution coverage since it varies based on research design, this value is considered relatively high, especially in large-number QCA studies, where lower coverage is more common. Solution PRI stands for the Proportional Reduction in Inconsistency at the solution level, which evaluates how consistently the identified configurations lead to the presence of the outcome, rather than its absence. In this study, the Solution PRI of 0.909 exceeds the 0.75 threshold, indicating a strong, reliable solution with minimal contradictions. Thus, the high solution consistency, solution coverage, and Solution PRI confirm that the seven pathways are reliable and effectively explain the outcome with minimal contradictions.

**Figure 3:**
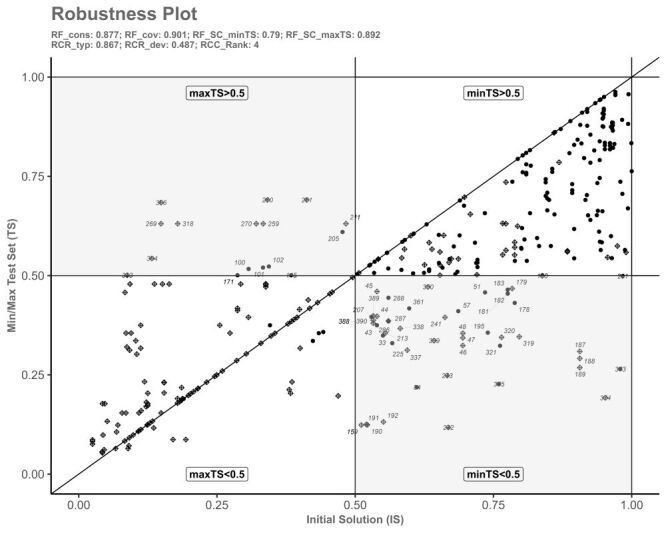
Robustness plot.

The robustness tests were conducted for both fit-oriented and case-oriented parameters. Sensitivity ranges were evaluated based on raw consistency and frequency thresholds. The fit-oriented raw consistency (RF_cons_) is 0.877, indicating a strong fit between the empirical data and the solution, while the coverage (RF_cov_) is 0.901, reflecting the proportion of cases the solution covers. Sensitivity tests showed stability across lower and upper thresholds of raw consistency (0.76–0.88) and frequency (n = 4), with RFSC__minTS_ and RFSC__maxTS_ values of 0.790 and 0.892, respectively, suggesting a stable solution across different parameter settings. In terms of cases-oriented measures, the ratio of robust typical cases (RCR_typ_) is 0.867, surpassing the ratio of robust deviant cases (RCR_dev_ = 0.487). This indicates that the solution fits better with typical cases compared to deviant cases. These robustness test results affirm the reliability of the findings, demonstrating that the solution is particularly stable when addressing typical cases. Finally, the RCC__Rank_ value of indicates that the complexity of the case analysis suggests a solution comprising a mix of ‘uncertain’ and ‘plausible’ cases, implying varying levels of robustness across different case types (**Figure 3**; Table S2 in the **Online Supplementary Document**).

## DISCUSSION

Our findings identified the key contributors and their potential complex interactions influencing life expectancy, particularly within the context of the COVID-19 pandemic. Determinants such as access to essential environmental quality, education attainment, economics prosperity, and public health capacity emerged as critical factors. These findings align with existing research emphasising the significant role of socioeconomic and environmental conditions in shaping population life expectancy globally [10–16,23]. Our findings suggest the need for a holistic approach to improving global life expectancy, one that integrates multiple policy areas such as enhancing access to essential infrastructure, education, economic growth, and the effectiveness of health systems. In addition, there isn’t a ‘one-size-fits-all’ solution; instead, different countries can follow different pathways to achieve improved life expectancy, depending on their unique socio-economic conditions and policy priorities.

Among the contributors to life expectancy, we found that access to electricity, represents environmental quality, emerged as the most influential in affecting life expectancy, reinforcing prior research that highlights the crucial role of basic infrastructure in promoting life expectancy [17,36–38]. Despite long-standing global efforts, energy poverty remains a significant issue. As of 2023, an estimated 745 million people worldwide still lacked access to electricity, with the problem being especially severe in low-income regions [39]. This challenge has been further compounded by global crises, including the COVID-19 pandemic and energy disruptions due to geopolitical events [39]. Therefore, continued investment in reliable and sustainable infrastructure, particularly in underserved areas, is critical not only for improving electricity access but also for ensuring clean water, internet, and other essential services. Policies could include subsidising renewable energy sources and developing public-private partnerships to expand grid coverage. Such investments are expected to raise living standards, reduce health disparities, thereby providing a foundational strategy for enhancing life expectancy globally.

Educational attainment, measured by mean years of schooling in this study, also is shown as an important factor influencing life expectancy, which is consistent with prior research [10–13]. Education is not only a social good but also a critical public health strategy with long-term effects on life expectancy. Education enhances individuals’ health literacy and awareness, improves access to health care resources, broadens socioeconomic opportunities, and equips individuals to make informed health decisions, thereby contributing to longer, healthier lives [10–13]. As of 2023, the global average years of schooling for adults aged 25 and over was approximately 6.57 years [40]. However, significant disparities exist across regions and socioeconomic groups. For instance, fewer than half of adults have completed primary education in sub-Saharan Africa, whereas in regions like Eastern Asia and Latin America, more than 75% of adults have achieved this milestone [40]. These education attainment disparities contribute directly to global inequalities in life expectancy. Our findings suggest that increasing educational opportunities, especially in underserved areas, have potential to help public health improvements. To address these disparities, targeted interventions should prioritise public investment in such as infrastructure, teacher training, resource allocation, and transportation, especially at the early education stages in underprivileged regions [41]. These efforts would not only help close the education gap but also foster a more educated and health-conscious population, contributing to long-term improvements in life expectancy.

Our study also revealed that economics prosperity, as measured by GNI *per capita*, is a crucial determinant of life expectancy, aligning with the well-established positive correlation between economic prosperity and longevity [14,42]. The disparity between high-income and low-income countries has widened in recent years. According to the 2023 World Bank classification, high-income countries like the USA, Germany, and Japan report a GNI *per capita* exceeding 13 205 USD, while low-income nations such as Afghanistan, Chad, and Malawi have a GNI *per capita* of 1085 USD or less [40]. These economic disparities significantly affect health, education, and living standards. Countries with higher GNI *per capita* generally provide better access to health care, education, and other essential services, contributing to longer life expectancy and improved quality of life. Additionally, our findings indicate that the percentage of the urban population and the measles immunisation rate are influential drivers of life expectancy, echoing the findings of some prior studies [21–23]. A higher proportion of urban residents often correlates with better access to health care facilities, sanitation, and education [43], all of which contribute to improved health outcomes and longer life expectancy through more developed health care infrastructure. Measles immunisation rates is a key indicator of public health system effectiveness. Higher vaccination coverage significantly reduces child mortality and prevents outbreaks of infectious diseases, thereby directly contributing to increased life expectancy [23]. To improve life expectancy, efforts should be focused on fostering economic development, expanding access to urban-quality living standards in rural areas, and enhancing health care system effectiveness through stronger immunisation programmes.

The key finding of this study is that improving life expectancy requires a holistic, multi-faceted approach due to the complex interplay of factors, including environment quality, education attainment, economics prosperity, urban development, and public health capacity. By addressing these interconnected areas and ongoing challenges, policy interventions can lead to more sustainable and equitable improvements in life expectancy, especially in underserved regions. Policy interventions that simultaneously target these factors are essential to achieving long-term health gains and reducing disparities across populations.

### Study limitations

Our study, while providing valuable insights, has several limitations that must be acknowledged. First, although our data sources represent multiple global data sets, variations in data collection methodologies across these sources may lead to inconsistencies in measurement and reporting. Additionally, we filtered the data to include only countries with complete and reliable data across all variables, resulting in the inclusion of 130 countries. While this stepwise approach minimised the impact of missing data and ensured consistency in the analysis, it may have excluded countries that are underrepresented in global data sets, potentially introducing selection bias and limiting the generalisability of our findings. Next, our analysis spans only three years (2020–2022), a period significantly influenced by the COVID-19 pandemic. While this three-year window may not fully capture long-term trends, it introduces the possibility of bias due to the extraordinary global context during this time. Last but not least, the QCA method, while effective for identifying complex causal pathways, may be sensitive to calibration decisions. Different thresholds or alternative approaches could yield different results. The reliability test results suggest that there is room for deeper exploration of the ‘uncertain’ and ‘plausible’ cases in future studies. Therefore, caution should be exercised in generalising our findings to other contexts.

## CONCLUSIONS

Using the qualitative comparative analysis method, this study contributes to the ongoing global discourse on life expectancy by identifying key determinants that have persisted during and after the COVID-19 pandemic. Access to electricity emerged as a necessary condition, underscoring the need for governments and organisations to prioritise expanding reliable electricity access, particularly in underserved areas, through investments in renewable energy and infrastructure development. Other factors, including mean years of schooling, immunisation rates, GNI *per capita*, and urban population, played critical roles in different pathways. Expanding access to education can be achieved through such as conditional cash transfer programmes, investments in teacher training, and the development of schools in rural areas. Strengthening health care systems requires improving vaccine delivery infrastructure, providing public education to enhance vaccine acceptance, and increasing incentives for immunisation efforts. Additionally, fostering job creation and promoting inclusive economic growth are essential to ensuring equitable access to health care, housing, and sanitation. Furthermore, our study suggests the need for integrated policies that are tailored to each country’s unique socio-economic contexts to promote sustainable and equitable health outcomes.

## Additional material


Online Supplementary Document

## References

[1] Robine JM Ritchie K Healthy life expectancy: Evaluation of global indicator of change in population health. BMJ. 1991;302:457–60. 1825931 10.1136/bmj.302.6774.457 PMC1669345

[2] Murray CJ Salomon JA Mathers C A critical examination of summary measures of population health. Bull World Health Organ. 2000;78:981–94. 10994282 PMC2560826

[3] Cao X Hou Y Zhang X Xu C Jia P Sun X A comparative, correlate analysis and projection of global and regional life expectancy, healthy life expectancy, and their GAP: 1995-2025. J Glob Health. 2020;10:020407. 33110572 10.7189/jogh.10.020407 PMC7568920

[4] Aksan A-M Chakraborty S Life expectancy across countries: Convergence, divergence and fluctuations. World Dev. 2023;168:106263.

[5] Liou L Joe W Kumar A Subramanian S Inequalities in life expectancy: An analysis of 201 countries, 1950–2015. Soc Sci Med. 2020;253:112964. 32247943 10.1016/j.socscimed.2020.112964

[6] Heuveline P Global and national declines in life expectancy: An end-of-2021 assessment. Popul Dev Rev. 2022;48:31–50. 37325186 10.1111/padr.12477 PMC10270701

[7] Huang G Guo F Liu L Taksa L Cheng Z Tani M Changing impact of COVID-19 on life expectancy 2019–2023 and its decomposition: Findings from 27 countries. SSM Popul Health. 2023;25:101568. 38144442 10.1016/j.ssmph.2023.101568 PMC10746558

[8] Hajdu T Krekó J Tóth CG Inequalities in regional excess mortality and life expectancy during the COVID-19 pandemic in Europe. Sci Rep. 2024;14:3835. 38360870 10.1038/s41598-024-54366-5 PMC10869827

[9] Bonnet F Grigoriev P Sauerberg M Alliger I Mühlichen M Camarda C-G Spatial variation in excess mortality across Europe: A cross-sectional study of 561 regions in 21 countries. J Epidemiol Glob Health. 2024;14:470–9. 38376764 10.1007/s44197-024-00200-0 PMC11176282

[10] van Baal P Peters F Mackenbach J Nusselder W Forecasting differences in life expectancy by education. Popul Stud (Camb). 2016;70:201–16. 27052447 10.1080/00324728.2016.1159718

[11] Meara ER Richards S Cutler DM The gap gets bigger: Changes in mortality and life expectancy, by education, 1981–2000. Health Aff (Millwood). 2008;27:350–60. 18332489 10.1377/hlthaff.27.2.350 PMC2366041

[12] IHME-CHAIN Collaborators Effects of education on adult mortality: A global systematic review and meta-analysis. Lancet Public Health. 2024;9:e155–65. 38278172 10.1016/S2468-2667(23)00306-7 PMC10901745

[13] Bilal U Hessel P Perez-Ferrer C Michael YL Alfaro T Tenorio-Mucha J Life expectancy and mortality in 363 cities of Latin America. Nat Med. 2021;27:463–70. 33495602 10.1038/s41591-020-01214-4 PMC7960508

[14] Mackenbach JP Looman CW Life expectancy and national income in Europe, 1900-2008: An update of Preston’s analysis. Int J Epidemiol. 2013;42:1100–10. 23920140 10.1093/ije/dyt122

[15] Miladinov G Socioeconomic development and life expectancy relationship: Evidence from the EU accession candidate countries. Genus. 2020;76:2.

[16] Zare H Gaskin DJ Anderson G Variations in life expectancy in Organization for Economic Co-operation and Development countries–1985–2010. Scand J Public Health. 2015;43:786–95. 26261191 10.1177/1403494815597357

[17] Saidmamatov O Saidmamatov O Sobirov Y Marty P Ruzmetov D Berdiyorov T Nexus between life expectancy, co2 emissions, economic development, water, and agriculture in aral sea basin: Empirical assessment. Sustainability. 2024;16:2647.

[18] Niu S Jia Y Wang W He R Hu L Liu Y Electricity consumption and human development level: A comparative analysis based on panel data for 50 countries. Int J Electr Power Energy Syst. 2013;53:338–47.

[19] Popoola OT Population growth and life expectancy in Nigeria: Issues and further considerations. Hum Soc Sci Res. 2018;1:30.

[20] Rau R Schmertmann CP District-level life expectancy in Germany. Dtsch Arztebl Int. 2020;117:493–9. 33087229 10.3238/arztebl.2020.0493 PMC7588608

[21] Singh GK Siahpush M Widening rural–urban disparities in life expectancy, US, 1969–2009. Am J Prev Med. 2014;46:e19–29. 10.1016/j.amepre.2013.10.017 24439358

[22] Abrams LR Myrskylä M Mehta NK The growing rural–urban divide in US life expectancy: Contribution of cardiovascular disease and other major causes of death. Int J Epidemiol. 2022;50:1970–8. 10.1093/ije/dyab158 34999859 PMC8743112

[23] van Wijhe M McDonald SA De Melker HE Postma MJ Wallinga J Effect of vaccination programmes on mortality burden among children and young adults in the Netherlands during the 20th century: A historical analysis. Lancet Infect Dis. 2016;16:592–8. 10.1016/S1473-3099(16)00027-X 26873665

[24] Neumayer E HIV/AIDS and cross-national convergence in life expectancy. Popul Dev Rev. 2004;30:727–42. 10.1111/j.1728-4457.2004.00039.x

[25] Dahlgren G Whitehead M The Dahlgren-Whitehead model of health determinants: 30 years on and still chasing rainbows. Public Health. 2021;199:20–4. 10.1016/j.puhe.2021.08.009 34534885

[26] United Nations. Life expectancy at birth. New York City, USA: United Nations; 2007. Available: https://www.un.org/esa/sustdev/natlinfo/indicators/methodology_sheets/health/life_expectancy.pdf#:~:text=Purpose%3A%20The%20indicator%20measures%20how%20many%20years%20a,mortality%20conditions%20and%2C%20by%20proxy%2C%20of%20health%20conditions. Accessed: 20 September 2024.

[27] DataBank. Metadata glossary. n.d. Available: https://databank.worldbank.org/metadataglossary/all/series. Accessed: 20 September 2024.

[28] UIS. Background information on education statistics in the UIS database. Montreal, Canada: UNESCO Institute for Statistics; 2021.

[29] Cascini F Failla G Gobbi C Pallini E Hui J Luxi W A cross-country comparison of Covid-19 containment measures and their effects on the epidemic curves. BMC Public Health. 2022;22:1765. 10.1186/s12889-022-14088-7 36115936 PMC9482299

[30] Della Porta D. Comparative analysis: Case-oriented versus variable-oriented research. In: Della Porta D, Keating M, editors. Approaches and methodologies in the social sciences. Cambridge, UK: Cambridge University Press; 2008. p. 198-223.

[31] Schneider CQ Wagemann C Standards of good practice in qualitative comparative analysis (QCA) and fuzzy-sets. Comp Sociol. 2010;9:397–418. 10.1163/156913210X12493538729793

[32] Thomann E Maggetti M Designing research with qualitative comparative analysis (QCA): Approaches, challenges, and tools. Sociol Methods Res. 2020;49:356–86. 10.1177/0049124117729700

[33] Mello PA. Qualitative comparative analysis: An introduction to research design and application. Washington, DC, USA: Georgetown University Press; 2021.

[34] Oana IE, Schneider CQ, Thomann E. Qualitative comparative analysis using R: A beginner's guide. Cambridge, UK: Cambridge University Press; 2021.

[35] Mello PA, Ragin CC. Measures of fit. In: Mello PA, editor. Qualitative comparative analysis: Research design and application. Washington, DC, USA: Georgetown University Press; 2019.

[36] Udoumoh E Subeno T William U Assessment of the impact of some socio-economic variables on worldwide average life expectancy: A logit approach. Asian J Probab Stat. 2023;21:29–36. 10.9734/ajpas/2023/v21i1454

[37] Njiru CW Letema SC Energy poverty and its implication on standard of living in Kirinyaga, Kenya. J Energy. 2018;2018:3196567. 10.1155/2018/3196567

[38] Acheampong AO Erdiaw-Kwasie MO Abunyewah M Does energy accessibility improve human development? Evidence from energy-poor regions. Energy Econ. 2021;96:105165. 10.1016/j.eneco.2021.105165

[39] Yolcan OO World energy outlook and state of renewable energy: 10-Year evaluation. Innov Green Dev. 2023;2:100070. 10.1016/j.igd.2023.100070

[40] UNDP. Human development report 2023/2024. New York, New York, USA: United Nations Development Programme; 2024.

[41] Keller KR Investment in primary, secondary, and higher education and the effects on economic growth. Contemp Econ Policy. 2006;24:18–34. 10.1093/cep/byj012

[42] Islam MS Mondal MNI Tareque MI Rahman MA Hoque MN Ahmed MM Correlates of healthy life expectancy in low-and lower-middle-income countries. BMC Public Health. 2018;18:476. 10.1186/s12889-018-5377-x 29642879 PMC5896094

[43] Čábelková I Gardanova Z Neimatov E Esaulov V Spatial accessibility assessment to healthcare facilities: Urban and rural areas. E3S Web of Conferences. 2021;301:02004. 10.1051/e3sconf/202130102004

