# Supplementary material for: Unravelling the determinants of life expectancy during and after the COVID-19 pandemic: a qualitative comparative analysis
Source: J Glob Health. 2025 Mar 28;15:04126. doi: 10.7189/jogh.15.04126 (PMC11950900; doi:10.7189/jogh.15.04126)
Supplement: Online Supplementary Document [file jogh-15-04126-s001.pdf]

Supplement to: Li X, Zhu JL, Park JM, Mitchell J. Unravelling the determinants of life expectancy during and after the COVID-19 pandemic: a qualitative comparative analysis. J Glob Health. 2025;15:04126.

Qualitative Comparative Analysis Calibration

The Significance of Calibration

Calibration is a critical step in Qualitative Comparative Analysis (QCA) as it transforms raw data into meaningful fuzzy set memberships, which are essential for identifying causal patterns. Figure A2 shows the calibration plots in our study. The significance of the calibration process includes the following points:

First, calibration converts raw, continuous data into fuzzy set memberships, assigning each case (an observation or unit of analysis) a score between 0 and 1. Continuous raw data often lacks the direct interpretability required for QCA, but calibration addresses this by indicating the degree to which a case belongs to a particular set. This transformation defines key threshold: full membership, crossover point, and full non-membership, providing empirical boundaries for set membership. For example, in the plot for Gross National Income (GNI) per Capita, a country with a GNI of $50,000 might receive a membership score close to 1, indicating full membership, while a country with a GNI of $10,000 might receive a membership score near 0.5, reflecting partial membership. Similarly, the calibration plot for Life Expectancy demonstrates how cases transition from non-membership (low life expectancy) to full membership (high life expectancy), highlighting the importance of these boundaries.

In addition, calibration standardizes variables, enabling comparison of diverse conditions measured on different scales. For example, Access to Electricity (%) is measured on a 0–100 scale, while Population Growth (%) may include negative values. Calibration

converts both into a common scale (0–1), making them directly comparable within the QCA framework.

Furthermore, using logistic transformation, calibration captures the nonlinear relationships between raw data and fuzzy set memberships. By adjusting the steepness parameter, researchers can tailor the calibration process to match the characteristics of each variable. For instance, the calibration plot for Prevalence of HIV shows a gradual increase in membership scores as raw values rise, capturing the nuanced relationship between prevalence rates and set membership. In contrast, a steeper transition can be observed in the plot for Access to Electricity, where cases with nearly 100% access rapidly transition to full membership, clearly differentiating them from cases with lower access rates. This ensures that membership scores are both analytically robust and reflective of the data's nuances.

Last but not least, calibration minimizes subjectivity by relying on empirical thresholds and statistical methods. Figure 1 demonstrates how thresholds are derived from the data (e.g., percentiles, medians) and consistently applied across all cases, ensuring that results are replicable and robust.

In summary, calibration is essential in QCA as it bridges the gap between raw quantitative data and qualitative set-theoretic analysis. By defining thresholds, applying logistic transformations, and visualizing results, calibration ensures that the analysis is both rigorous and interpretable.

Calibration Process

The concept of calibration is most often associated with fuzzy sets. In this study, our calibration process relies on the statistical methods provided by the R "QCA 3.22" package, which uses data-driven thresholds derived from the empirical distribution of variables. This

approach ensures an objective and consistent transformation of raw data into fuzzy set memberships. Specifically, the calibration process employs the "calibrate()" function to standardize data into set memberships.

Step 1: Selection of calibration thresholds: The calibration process begins by selecting three key thresholds for each variable: full membership (1): the value at which cases are considered to fully belong to the set; crossover point (0.5): the value representing maximum ambiguity between membership and non-membership; and full non-membership (0): the value at which cases are considered to not belong to the set. These thresholds were determined based on the empirical distribution of the data, using statistical measures: percentiles. Specifically, the 95th percentile serves as the full membership threshold, the 50th percentile as the crossover point, and the 5th percentile as the full non-membership threshold.

Step 2: Application of the calibration function: The selected thresholds are then used in the calibration process. The "calibrate()" function is applied to transform raw values into fuzzy set membership scores for each variable. This function performs a logistic transformation of the data based on the selected thresholds, ensuring a smooth and consistent mapping of raw values to membership scores.

The formula used is:

$$M\ (x) = \frac{1}{1 + e^{-k(x - x_0)}}$$

Where:

- $M(x)$: Fuzzy set membership score
- x: Raw data value
- x_0: Crossover point (0.5 threshold)
- k: Steepness parameter controlling the transition between scores.

The logistic function ensures that: scores close to 1 indicate full membership; scores close to 0 indicate full non-membership; and scores around 0.5 represent maximum ambiguity (neither fully in nor out of the set).

Step 3: Generation of membership scores: After applying the calibration function, the output is a vector of membership scores for each case and variable. These scores range from 0 (full non-membership) to 1 (full membership), with intermediate values indicating partial membership. For instance, a case with a raw data value slightly above the crossover point will receive a membership score closer to 1, while a value below the crossover point will produce a score closer to 0.

In summary, this calibration process, transforming raw data into fuzzy set memberships, was conducted in a rigorous and transparent manner, providing a solid foundation for our study analyses.

Figure S1. Data Derivative

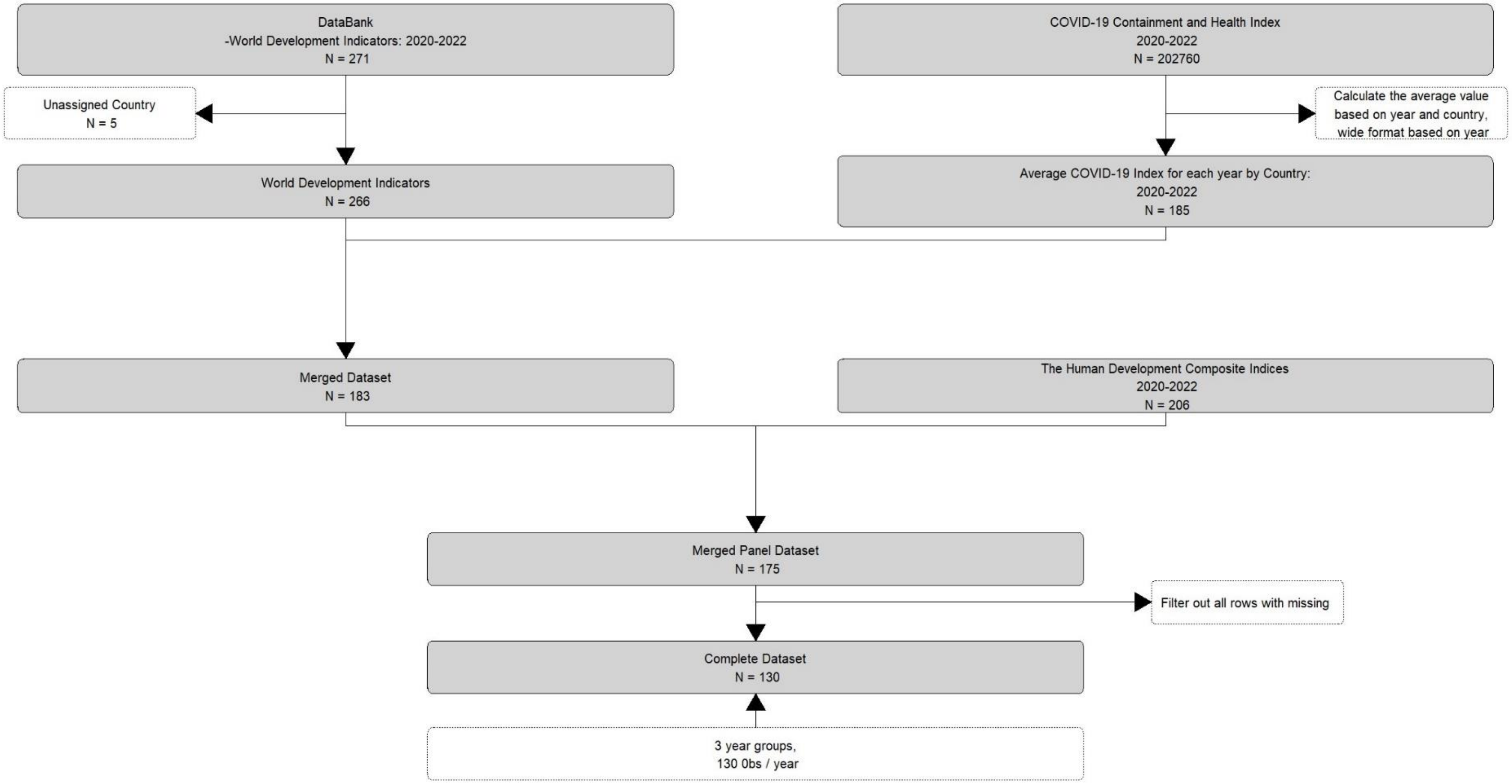

Figure S2. Variables' Calibration Plots

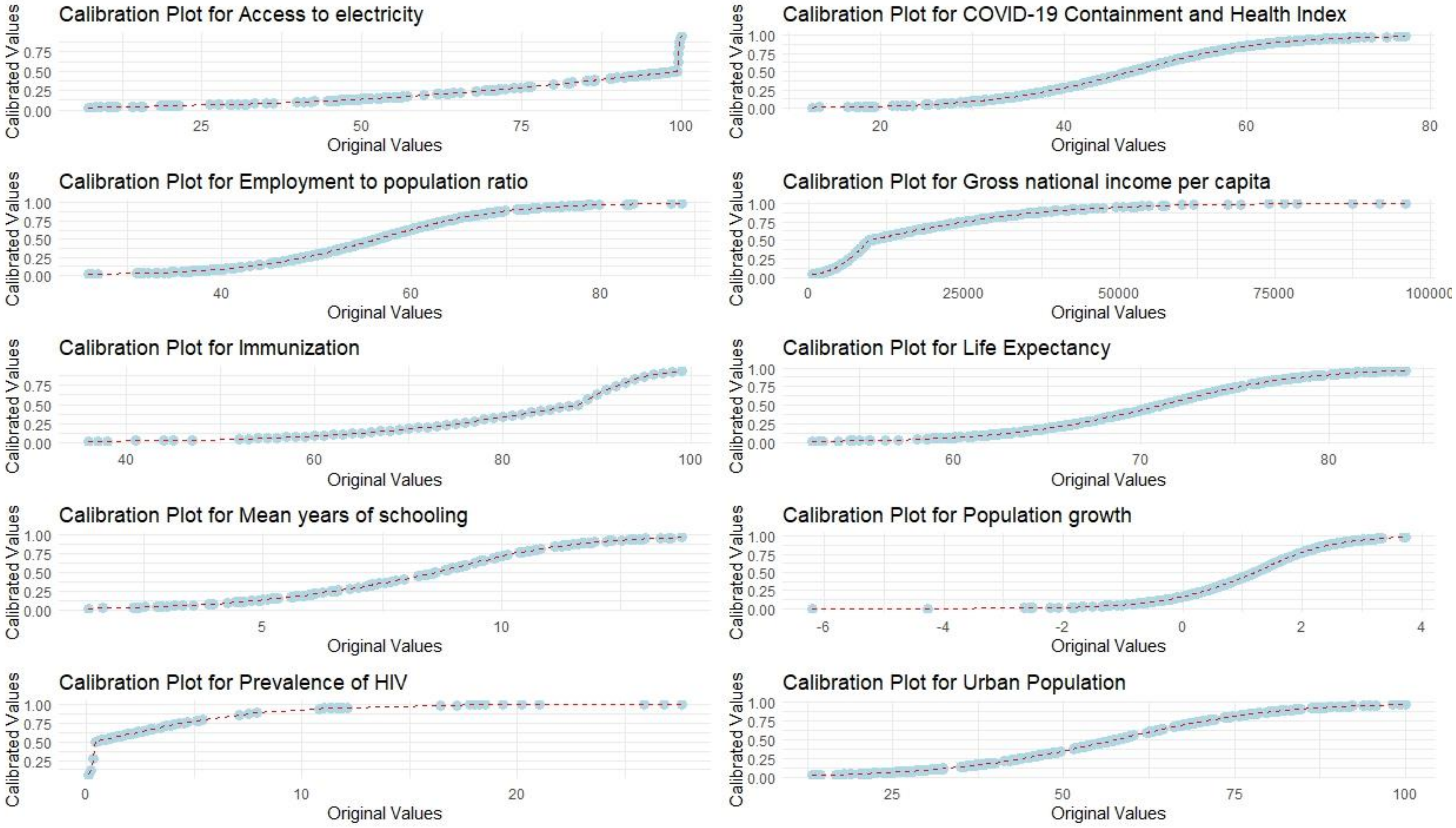

Table S1. QCA Calibration Results:

	Variable	Calibration		
		**Full membership**	**Crossover point**	**Full non-membership**
**Dependent Variable**	Life expectancy	82.267	71.078	58.961
**Independent Variables/Conditions**	Mean years of schooling	12.960	8.615	2.777
	Gross national income per capita	50,568.363	9,702.233	1,264.033
	Access to electricity (% of population)	100.000	99.400	19.225
	Population growth (annual %)	3.059	1.226	-0.989
	Employment to population ratio	75.815	56.452	35.993
	Urban Population (% of total population)	92.176	57.557	20.542
	Immunization, measles (% of children ages 12-23 months)	99.000	88.000	53.450
	Prevalence of HIV	11.355	0.400	0.100
	COVID-19 Containment and Health Index	68.710	47.233	25.040

Calibration thresholds: 0.95, 0.5, 0.05

Table S2. QCA Robustness Tests Results

**Sensitivity Ranges**				
Parameters	Raw consistency	Lower: 0.76	Threshold: 0.80	Upper: 0.88
	Frequency	Lower: 4	Threshold: 4	Upper: 4
**Robustness Parameters**				
Fit oriented	RF_{cons}: 0.877	RF_{cov}: 0.901	$RFSC_{\_minTS}$: 0.790	$RFSC_{\_maxTS}$: 0.892
Case oriented	RCR_{typ}: 0.867	RCR_{dev}: 0.487	$RCC_{\_Rank}$: 4	

RF_{cons} – raw frequency consistency, RF_{cov} – raw frequency coverage, $RFSC_{\_minTS}$ – raw frequency-set consistency minimum threshold setting, $RFSC_{\_maxTS}$ – raw frequency-set consistency maximum threshold setting, RCR_{typ} – raw coverage ratio (typical cases), RCR_{dev} – raw coverage ratio (deviant cases), RCC_Rank – raw coverage contribution ran
